# Socio-behavioral determinants of health-related quality of life among patients with type 2 diabetes: comparison between 2015 and 2018

**DOI:** 10.1186/s12902-024-01604-6

**Published:** 2024-05-20

**Authors:** Neda Izadi, Arman Shafiee, Mahdieh Niknam, Reza Yari-Boroujeni, Fereidoun Azizi, Parisa Amiri

**Affiliations:** 1 Research Center for Social Determinants of Health, Research Institute for Endocrine Sciences, Shahid Beheshti University of Medical Sciences, Tehran, Iran; 2 School of Medicine, Alborz University of Medical Sciences, Alborz, Iran; 3 Endocrine Research Center, Research Institute for Endocrine Sciences, Shahid Beheshti University of Medical Sciences, Tehran, Iran

**Keywords:** Socio-behavioral determinants, Health-related quality of life, Type 2 diabetes

## Abstract

**Introduction:**

Type 2 diabetes (T2D) is a common chronic disease that significantly affects an individual’s overall health and well-being. The aim of this study is to investigate the factors that influence the health-related quality of life (HRQoL) of patients with T2D.

**Methods:**

This study conducted using data from 6th phase (2015–2017) and 7th phase (2018–2022) of the Tehran Lipid and Glucose Study (TLGS). Data were collected through a combination of interviews, physical examinations, and laboratory tests. Quality of life questionnaire (SF-12) that consists of 12 questions was used to assess physical and mental health functioning. The generalized estimating equation model was used to assess the association between socio-behavioral factors and changes in HRQoL.

**Results:**

The study included 498 patients with T2D. The changes in HRQoL in patients with T2D followed a sex-specific pattern. Analysis of the physical component score (PCS) and the mental component score (MCS) showed a non-significant change in the total score during the three-year longitudinal study. However, the role physical (RP) of the PCS and the social functioning (SF) of the MCS showed a statistically significant change during this period. In addition, sex, body mass index (BMI), and having cardiovascular disease (CVD) and chronic kidney disease (CKD) showed a significant association with RP changes, and only job status showed a significant association with SF changes.

**Conclusions:**

By recognizing the sex-specific patterns in HRQoL changes and understanding the multifaceted nature of factors such as BMI, CVD and CKD, healthcare professionals can develop targeted interventions that go beyond traditional diabetes management.

## Introduction

Type 2 diabetes (T2D) is a common chronic disease that significantly affects an individual’s overall health and well-being [1]. It is characterized by insulin resistance and high blood glucose levels, which necessitates the use of various self-care practices [2]. According to reports from the International Diabetes Federation (IDF), the global prevalence of diabetes is estimated to be 9.3% (463 million people) in 2019, reaching 10.2% in 2030 and 10.9% in 2045 [3]. The prevalence of diabetes in different ethnic groups worldwide has been reported to be between 7.8% and 15.5% [4].

According to the World Health Organization (WHO), the overall prevalence of diabetes in Iran is 10.3%, with an estimated 9.6% in men and 11.1% in women [5]. Also, based on a national population-based survey conducted in four rounds using the STEPwise approach to risk factor surveillance (STEPS) for non-communicable disease (NCDs), the prevalence of diabetes in Iran has increased in the last 12 years (2004 to 2016) and stood at 8.4, 9, 11.1 and 13.2% respectively. The age- and sex-weighted prevalence of T2D among the 8,151 adult residents of Tehran was 16.7% and diabetes was found to be more prevalent in the eastern and central districts of Tehran [6]. In addition, there was a significant association between female gender, age over 40 years, living in the city, being in the third wealth quintile, waist-to-hip ratio, hypercholesterolemia, low‐density lipoprotein, hypertension, cardiovascular disease (CVD), stroke, and having health insurance with diabetes prevalence [6–8].

Individuals with T2D have an increased risk of developing various complications such as cardiovascular disease, neuropathy and retinopathy, which can significantly affect their quality of life [9]. However, effective management of T2D requires an understanding of the socio-behavioral determinants that influence health-related quality of life [10].

Health-related quality of life (HRQoL) encompasses an individual’s perception of their own health status, including physical, mental, and social dimensions [11]. Therefore, it is critical to explore the socio-behavioral determinants that influence quality of life in individuals with T2D. This understanding will facilitate the development of targeted interventions and support systems tailored to the specific needs and challenges of these individuals [12].

Socio-behavioral determinants include a range of factors that can either facilitate or hinder a person’s ability to effectively manage their diabetes and maintain a good quality of life. Socioeconomic factors such as income, education, and occupation play an important role in the quality of life among patients with T2D. They influence access to healthcare services, the availability of resources for self-care, and the ability to make informed health decisions [13].

Lifestyle behaviors, such as physical activity and smoking, also have a significant impact on the HRQoL of individuals with T2D. A healthy lifestyle is crucial for controlling blood glucose levels, managing weight, and reducing the risk of complications. However, cultural norms, personal beliefs, and environmental influences can either facilitate or hinder the adoption of healthy behaviors [14–16].

Understanding the socio-behavioral determinants of HRQoL in patients with T2D is essential for developing comprehensive approaches to support their well-being. By addressing factors such as socioeconomic status and lifestyle, healthcare professionals and policymakers can develop targeted interventions that promote positive health outcomes and improve the overall quality of life of patients with T2D. On the other hand, by comparing data from 2015 to 2018, the study can provide a longitudinal perspective on the socio-behavioral factors that affect HRQoL in patients with T2D. This may help to identify trends, areas of improvement or potential challenges in the management of diabetes-related quality of life over time. The aim of this study is to investigate the factors that influence health-related quality of life in patients with type 2 diabetes.

## Methods

### Study design and participants

The Tehran Lipid and Glucose Study (TLGS) is a population-based prospective study conducted in Tehran, Iran. The study population consists of residents of district 13 of Tehran, who were selected using a multistage cluster sampling method. The study design and sampling strategy have been described in detail elsewhere [17]. In brief, eligible participants were aged 3 years or older. A total of 15,005 individuals were enrolled in the study between 1999 and 2001, and followed up every three years thereafter. To date, seven phases of the study have been completed. This study conducted using data from 6th phase (2015–2017) and 7th phase (2018–2022) of the TLGS. In Phase VI, the HRQoL questionnaire was completed by 8,048 participants, and in Phase VII by 7,258 participants. After merging the data from both phases, the total number of overlapping cases was 5,137 individuals. Among these individuals, 508 were diagnosed with T2D in phase VI. After excluding the missing, data from 498 patients with T2D were included in the study.

### Data collection

Data were collected through a combination of interviews, physical examinations, and laboratory tests. Trained interviewers administered a standardized questionnaire to collect information on demographic characteristics (age, sex, educational level, marital status, and job status), lifestyle factors (smoking and physical activity), and medical history. Physical examinations were conducted by trained staff, who measured anthropometric measurements including height, weight, waist circumference (WC), and also blood pressure (BP). Body weight was measured with a digital scale while participants wore minimal clothing and no shoes. Body height was measured while participants stood in normal posture and without shoes. Leisure-time physical activity data were collected using the Iranian version of the Modifiable Activity Questionnaire (MAQ) [18]. The Iranian version of the MAQ has been shown to be highly reliable and moderately valid in the past [19]. Participants were asked to report the frequency and duration of activities performed in each domain over the previous week. Metabolic equivalent (MET) values were assigned to each activity, and total MET-minutes per week were calculated [20]. Fasting blood samples were collected from participants after an overnight fast of at least 12 h for measurement of lipid and glucose levels. All laboratory tests were conducted at the central laboratory of the TLGS, using standardized protocols and quality control procedures. Serum total cholesterol (TC), triglycerides (TG), high-density lipoprotein cholesterol (HDL-C), and low-density lipoprotein cholesterol (LDL-C) were measured using enzymatic methods. Fasting plasma glucose levels were measured using the glucose oxidase method.

### Measures

Participants were categorized as illiterate/primary, secondary/diploma, and academic. Smoking status was defined as current smoker, ex-smoker, and nonsmoker. Physical activity level was categorized as low (≤ 600 MET min/week) and moderate/high (> 600 MET min/week). Body mass index (BMI) was calculated using the formula weight (kg)/height^2^ (m). Hypertension defined as systolic BP > 140 mmHg and/or diastolic BP > 90 mmHg or medication use to treat hypertension (yes or no) [21]. In addition, diabetes was defined as: a fasting blood glucose level of 126 mg/dL or higher, a 2-hour blood glucose level of 200 mg/dL or higher or the use of anti-diabetic medication (yes or no) [22]. Cardiovascular disease defined as self-reported positive history of myocardial infarction and/or ischemic heart disease and/or stroke and/or ECG positive and/or medication use to treat CVD (yes or no). In chronic kidney disease (CKD), the Modification of Diet in Renal Disease (MDRD) formula was used to estimate the glomerular filtration rate (GFR) in ml/min/1.73 m² body surface area [23]. The abbreviated MDRD equation is as: GFR = 186 × (serum creatinine)^-1.154 × (age)^-0.203 × (0.742 for women).

Based on the estimated GFR values, patients were divided into two groups: Non-CKD with a GFR of 60 mL/min/1.73 m² or higher and CKD with a GFR of less than 60 mL/min/1.73 m² [24]. Dyslipidemia was defined as hypercholesterolemia, and/or hypertriglyceridemia, and/or hyper-LDL, and/or hypo-HDL based on the National Cholesterol Education Program Adult Treatment Panel III (NCEP ATP III) classification of lipid profile (yes or no) [25]. Diseases in the family history reflect a previous diagnosis of the named diseases in first-degree relatives.

### Health-related quality of life questionnaire (SF-12)

The SF-12 is the abridged practical version of the 36-item Short Form Health Survey. The SF-12 questionnaire version 2.0 (SF-12v2) is a widely used survey instrument that measures health-related quality of life across various domains. It consists of 12 questions that assess physical and mental health functioning. The questionnaire covers two main domains including physical component score (PCS) and mental component score (MCS) and also eight subdomains, including physical functioning (PF, 2 items), role limitations due to physical health problems or role physical (RP, 2 items), bodily pain (BP, 1 items), general health (GH, 1 items), vitality (VT, 1 items), social functioning (SF, 1 items), role limitations due to emotional problems or role emotional (RE, 2 items), and mental health (MH, 2 items). Each domain provides insight into different aspects of an individual’s well-being and overall quality of life [26, 27]. According to the study by Montazeri et al. (2011), the SF-12v2 is a reliable and valid measure of health-related quality of life among the Iranian population [28]. The scores for each domain of the SF-12v2 range from 0 (the worst) to 100 (the best).

### Statistical analysis

Descriptive statistics were used to summarize the baseline characteristics of the study population. Continuous variables were presented as means ± standard deviations or medians (interquartile range) for normally distributed and skewed variables, respectively. Categorical variables were reported as frequencies and percentages. The chi-square test was used to compare categorical variables, while the t-test or Mann-Whitney U test was used for continuous variables, as appropriate. In addition, paired t-test was used to examine significant changes in different domains over the three-year period. The generalized estimating equation (GEE) model was used to assess the association between socio-behavioral factors and changes in RP and SF of HRQoL. All significant variables of the univariable model were included in the multivariable analysis. The analyses were performed using STATA version 14. A two-sided p-value < 0.05 was considered statistically significant.

### Ethical considerations

The TLGS was approved by the ethics committee of the Research Institute for Endocrine Sciences, Shahid Beheshti University of Medical Sciences. All participants provided written informed consent before enrollment in the study.

## Results

### Baseline characteristics

**Fig. 1:**
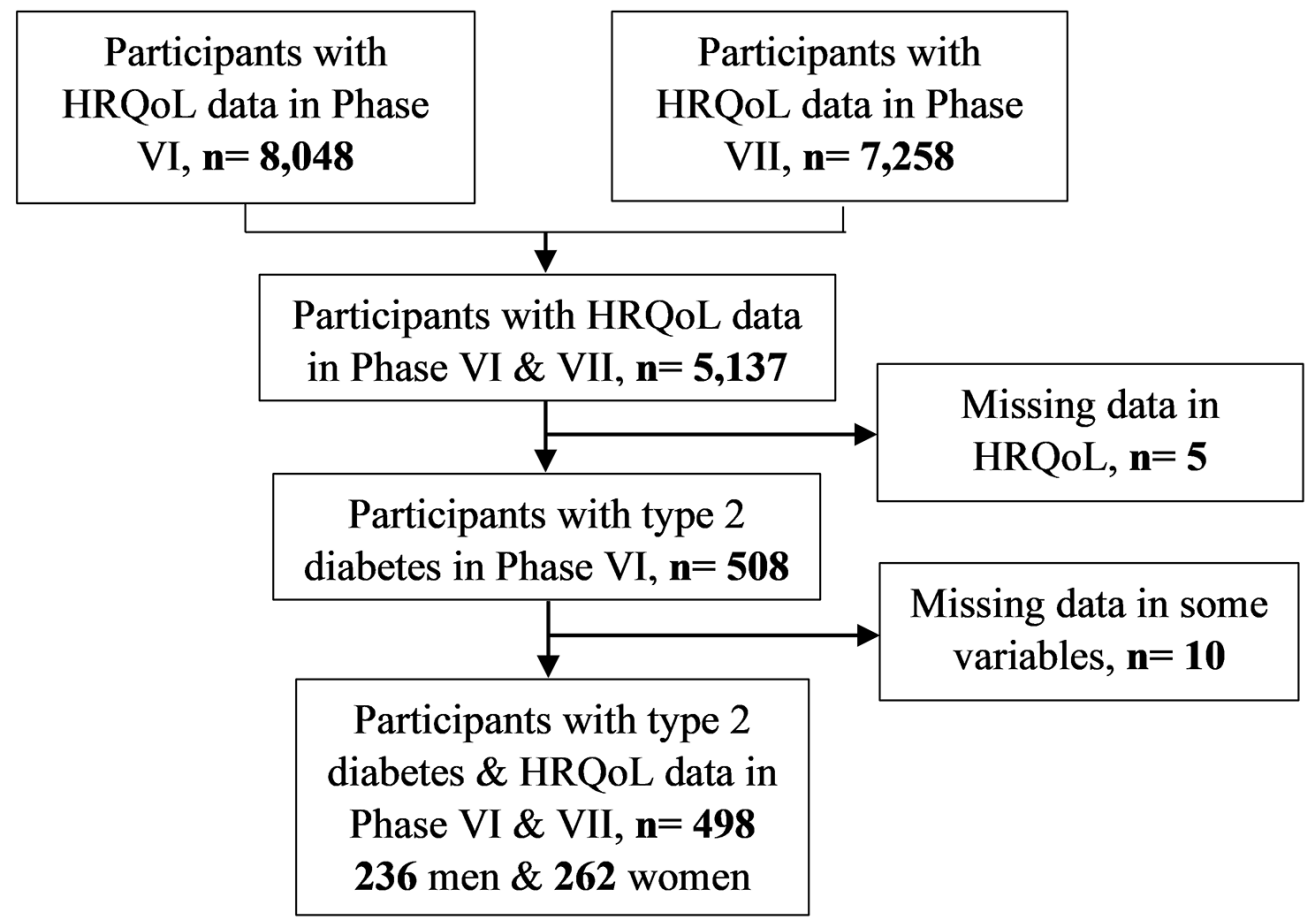
Flowchart of the study participants

The study included 498 patients with T2D (Fig. 1), with a mean age of 55.75 ± 10.31 years and an age range of 21 to 87 years. The mean age of male patients (56.84 years) was significantly higher than that of female patients (54.77 years) (*P* = 0.025). 52.61% (262) of patients were female (sex ratio: 1.11 women/men). Among the patients, 22.29% were illiterate. The frequency of illiteracy was significantly higher in women (27.48%) compared to men (16.53%). The majority of patients were married (86.92%), with a higher percentage in males. Of the male patients, 62.13% were employed, while only 7.63% of female patients were employed.

Regarding smoking habits, 9.48% of patients were current smokers, with a significantly higher frequency in men than in women (17.02% vs. 2.68%) (*P* < 0.001). Approximately 47.94% of patients had high physical activity, with no significant difference between men and women (*P* = 0.175). The mean BMI among patients was 29.85 ± 5.00 kg/m², and was significantly higher in women than in men. However, mean WC was similar in men and women. In regard to blood pressure, mean systolic and diastolic blood pressure was significantly higher in men than in women. Although mean or median lipid profile levels were higher in women with T2D than in men, the difference was significant only for HDL-C. The prevalence of hypertension, cardiovascular disease, chronic kidney disease, and dyslipidemia in patients with T2D was 27.71%, 24.50%, 35.69%, and 92.96%, respectively. A family history of diabetes, hypertension, and cardiovascular disease was reported by 15.67%, 16.74%, and 3.02% of patients, respectively. In addition, family history of diabetes was significantly higher in women than in men (20.08% vs. 10.62%) (*P* = 0.004) (Table 1).

### Changes in health-related quality of life scores and their domains

**Fig. 2:**
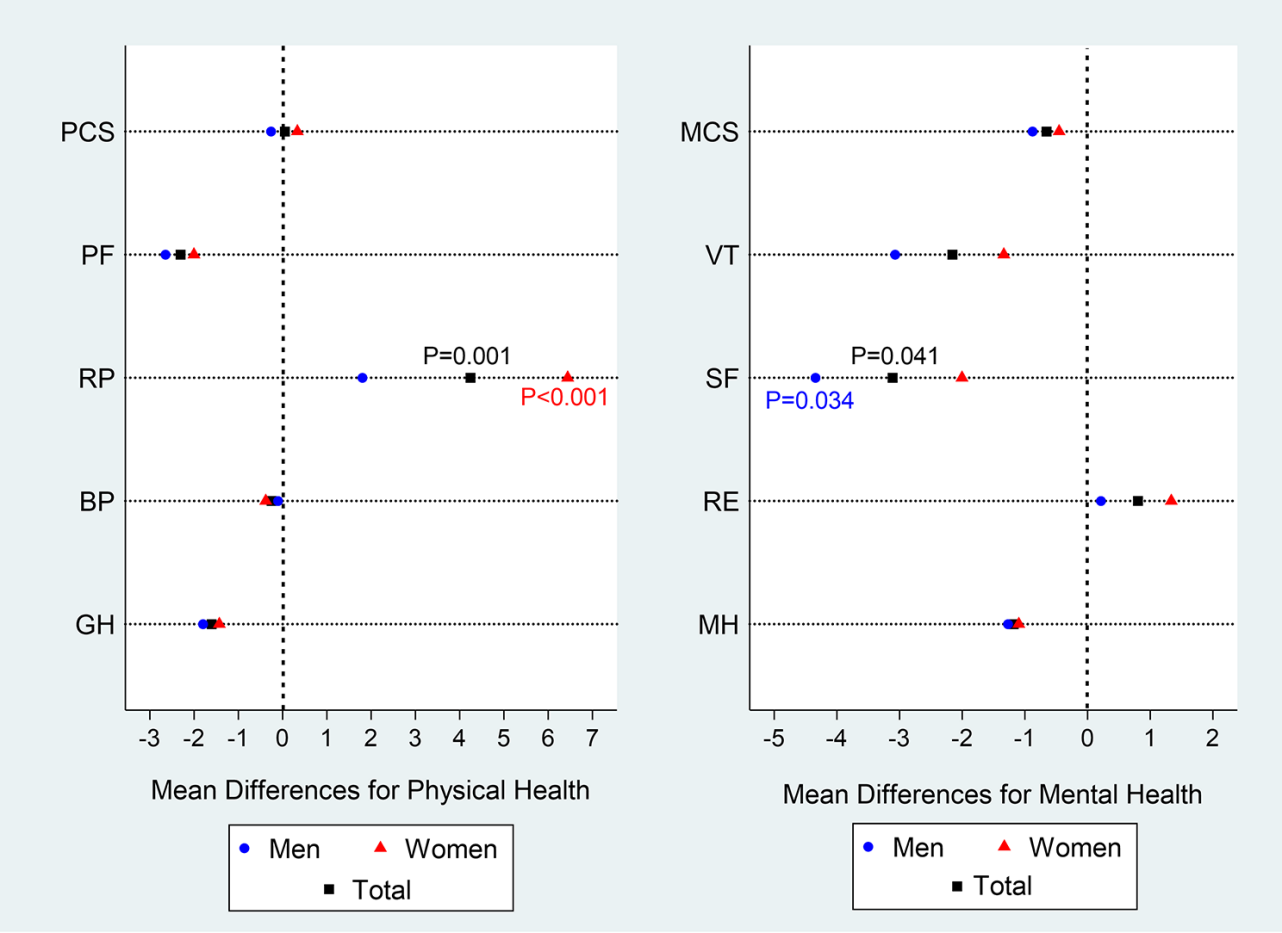
The mean difference of health-related quality-of-life scores (based on phases VI and VII data) in patients with diabetes by sex. PCS = Physical component score; PF = Physical functioning; RF = Role physical; BP = Bodily pain; GH = General health; MCS = Mental component score; VT = Vitality; SF = Social functioning; RE = Role emotional; MH = Mental health; Based on paired t-test

In phase VI, the mean PCS and MCS of health-related quality of life in patients with at least a three-year history of T2D were 46.14 ± 8.52 and 69.87 ± 23.16, respectively. The PCS was significantly higher in male patients than in female patients. After a period of three years, the mean PCS increased slightly in women and decreased slightly in men, but this difference was not statistically significant. The mean scores of all PCS domains, including PF, RP, BP, and GH, were significantly higher in men than women in both phases. In phase VI, the highest mean PCS domains was found in physical functioning (80.42 ± 26.07) and the lowest in general health (38.60 ± 20.17). However, in phase VII, the highest mean was found in role physical (80.14 ± 23.75), and the lowest in general health (36.99 ± 18.437). When examining changes over the three-year period, patients showed decreases in all PCS domains except role physical, but these were not statistically significant. The only significant increased observed in all patients and women were in the role physical domain (Tables 2 and Fig. 2).

In addition, after the three-year period, all patients with T2D, regardless of sex, showed a very small, statistically non-significant decrease in mean mental component score. Mean scores of all MCS domains, including VT, SF, RE, and MH, were significantly higher in men than in women in both phases (with the exception of SF in phase VII). The highest mean scores of MCS domains in both phases were observed in social functioning (81.32 ± 25.81 and 78.21 ± 29.79), and the lowest in vitality (63.65 ± 27.86 and 61.49 ± 25.16, respectively). When examining changes in MCS domains over the three-year period, patients showed decreases in all domains except role emotional, but these were not statistically significant. Only in social functioning was significant decline in all patients and men (Tables 2 and Fig. 2).

In general, changes in HRQoL among patients with T2D followed a sex-specific pattern. The most significant decline in social functioning was observed in men. Conversely, women with T2D showed significant improvement in the role physical during the three-year assessment period. In other words, the women showed improved physical compatibility in performing tasks and daily activities.

### Socio-behavioral determinants of HRQoL in patients with T2D

#### Role physical

When examining the association between socio-behavioral variables and role physical in all patients with T2D using a univariable model, sex, academic education, job status, smoking status, BMI, having CVD and CKD, and a family history of CVD had a significant association with RP changes. However, in multivariable regression and after adjustment for significant variables in the univariable model, sex, BMI, and having CVD and CKD showed a significant association with RP changes. In female patients, RP decreased by an average of 10.89 compared with men (*P* < 0.001), and when patients’ BMI increased by 1 kg/m^2^, RP decreased by 0.51 (*P* = 0.002). In patients with CVD and CKD, RP was lower than in other patients by an average of 4.03 (*P* = 0.024) and 3.33 units (*P* = 0.038), respectively.

In women, academic education, BMI, and the presence of CVD and CKD showed a significant association with role physical changes. However, in the multivariable model, only BMI and CKD remained significantly associated with RP changes. When women’s BMI increased by 1 kg/m^2^, RP decreased by an average of 0.84 units (*P* < 0.001). In addition, RP was an average 5.59 units lower in women with CKD than in other patients (*P* = 0.013) (Table 3).

#### Social functioning

In association between socio-behavioral variables and social functioning in all patients with T2D, sex, job status, having CVD and family history of diabetes, hypertension and CVD had a significant association with SF changes. However, in multivariable regression and after adjustment, only job status showed a significant association with SF changes. In unemployed patients, SF decreased by an average of 5.43 compared with others (*P* = 0.028).

In men, marital status and family history of diabetes and hypertension showed a significant association with social functioning changes. In addition, in the multivariable model, marital status and family history of hypertension significantly associated with SF changes. In unmarried patients, SF decreased by an average of 12.86 units compared with married patients (*P* = 0.048). In addition, SF was an average 8.79 units lower in men with family history of hypertension than in other patients (*P* = 0.021) (Table 4).

**Table 1:** Frequency and distribution of different variables at baseline (phase VI) in patients with diabetes by sex

Variables		Total (*n* = 498)	Men (*n* = 236)	Women (*n* = 262)	*P*-value^*^
		*N* (%)			
**Age (year)†**		55.75 (10.31)	56.84 (10.64)	54.77 (9.93)	**0.025** ^**^
**Educational level**					
	Illiterate/primary	111 (22.29)	39 (16.53)	72 (27.48)	**0.001**
	Secondary/diploma	290 (58.23)	137 (58.05)	153 (58.40)	
	Academic	97 (19.48)	60 (25.42)	37 (14.12)	
**Marital status**					
	Married	432 (86.92)	225 (95.34)	207 (79.31)	**< 0.001**
	Unmarried	65 (13.08)	11 (4.66)	54 (20.69)	
**Job status**					
	Employed	166 (33.40)	146 (62.13)	20 (7.63)	**< 0.001**
	Unemployed	331 (66.60)	89 (37.87)	242 (92.37)	
**Smoking status**					
	Current smoker	47 (9.48)	40 (17.02)	7 (2.68)	**< 0.001**
	Ex-smoker	58 (11.69)	56 (23.83)	2 (0.77)	
	Non**-**smoker	391 (78.83)	139 (59.15)	252 (96.55)	
**Leisure time physical activity**					
	Low (≤ 600 MET min/week)	139 (52.06)	58 (47.54)	81 (55.86)	0.175
	Moderate/High (> 600 MET min/week)	128 (47.94)	64 (52.46)	64 (44.14)	
**Anthropometric indices†**					
	Body Mass Index, kg/m^2^	29.85 (5.00)	28.68 (4.28)	30.92 (5.36)	**< 0.001** ^**^
	Waist circumference, cm	99.74 (10.65)	100.06 (10.57)	99.45 (10.73)	0.524^**^
**Blood pressure†**					
	Systolic, mmHg	123.11 (17.56)	125.91 (15.47)	120.57 (18.94)	**< 0.001** ^**^
	Diastolic, mmHg	79.94 (9.77)	82.48 (9.28)	77.64 (9.65)	**< 0.001** ^**^
**Lipid profile†**					
	Total cholesterol, mg/dl	181.50 (44.43)	177.47 (44.61)	185.10 (44.04)	0.056^**^
	Triglyceride, mg/dl††	151 (109.5)	149 (119)	151.5 (100)	0.921^**£**^
	LDL-C, mg/dl	102.08 (37.05)	100.46 (36.59)	103.51 (37.47)	0.368^**^
	HDL-C, mg/dl	44.64 (10.80)	41.40 (10.47)	47.53 (10.27)	**< 0.001** ^**^
**Comorbidities (yes)**					
	Hypertension	138 (27.71)	74 (31.36)	64 (24.43)	0.085
	Cardiovascular disease	122 (24.50)	58 (24.58)	64 (24.43)	0.969
	Chronic kidney disease	177 (35.69)	60 (25.64)	117 (44.66)	**< 0.001**
	Dyslipidemia	462 (92.96)	219 (39.19)	243 (92.75)	0.847
**Family history (yes)**					
	Diabetes	76 (15.67)	24 (10.62)	52 (20.08)	**0.004**
	Hypertension	81 (16.74)	33 (14.41)	48 (18.82)	0.194
	Cardiovascular disease	15 (3.02)	5 (2.13)	10 (3.83)	0.269

^†^Mean (Standard deviation); ^††^Median (Interquartile range); **Unmarried**: Single, divorce, widow; **Unemployed**: Student, housewife, other; **LDL-C**: Low-Density Lipoprotein Cholesterol; **HDL-C**: High-Density Lipoprotein Cholesterol; ^*^Based on Chi-square; ^**^Based on T-test; ^**£**^Based on Mann-Whitney U test

**Table 2:** The distribution of health-related quality-of-life scores in patients with diabetes by sex

Domains		Phase VI (2015–2017)			*P*-value^*^	Phase VII (2018–2022)			*P*-value^*^
		Total (*n* = 498)	Men (*n* = 236)	Women (*n* = 262)		Total (*n* = 498)	Men (*n* = 236)	Women (*n* = 262)	
		Mean ± SD							
**PCS**		46.14 ± 8.52	48.38 ± 6.86	44.12 ± 9.34	**< 0.001**	46.19 ± 8.55	48.11 ± 7.50	44.46 ± 9.06	**< 0.001**
	**Physical functioning**	80.42 ± 26.07	87.60 ± 19.50	73.95 ± 29.38	**< 0.001**	78.11 ± 26.87	84.95 ± 22.98	71.94 ± 28.62	**< 0.001**
	**Role physical**	75.90 ± 24.35	84.21 ± 20.26	68.41 ± 25.32	**< 0.001**	80.14 ± 23.75	86.01 ± 20.89	74.85 ± 24.93	**< 0.001**
	**Bodily pain**	77.05 ± 25.11	83.58 ± 21.70	71.18 ± 26.52	**< 0.001**	76.80 ± 28.04	83.47 ± 24.23	70.80 ± 29.87	**< 0.001**
	**General health**	38.60 ± 20.17	40.67 ± 20.70	36.73 ± 19.52	**0.029**	36.99 ± 18.43	38.87 ± 18.61	35.30 ± 18.14	**0.030**
**MCS**		48.98 ± 11.54	50.73 ± 10.61	47.39 ± 12.12	**0.001**	48.32 ± 10.84	49.86 ± 10.35	46.94 ± 11.09	**0.002**
	**Vitality**	63.65 ± 27.86	67.69 ± 26.73	60.01 ± 28.40	**0.002**	61.49 ± 25.16	64.61 ± 25.12	58.68 ± 24.92	**0.008**
	**Social functioning**	81.32 ± 25.81	84.95 ± 24.21	78.05 ± 26.80	**0.002**	78.21 ± 29.79	80.61 ± 28.67	76.04 ± 30.65	0.087
	**Role emotional**	75.47 ± 24.18	80.29 ± 22.44	71.13 ± 24.90	**< 0.001**	76.28 ± 24.69	80.50 ± 24.10	72.47 ± 24.64	**< 0.001**
	**Mental health**	69.87 ± 23.16	75.21 ± 21.16	65.07 ± 23.85	**< 0.001**	68.69 ± 22.94	73.94 ± 21.36	63.97 ± 23.33	**< 0.001**

**PCS =** Physical component score; **MCS =** Mental component score; *Based on T-test

**Table 3:** The association between socio-behavioral factors and changes of role physical (RP) using generalized estimating equation (GEE)

Variables		Total (*n* = 498)						Women (*n* = 262)					
		β†	SE	*P*-value	β††	SE	*P*-value	β†	SE	*P*-value	β††	SE	*P*-value
**Age (year)**		-0.05	0.08	0 0.528	**-**			-0.15	0.11	0.188	-		
**Sex (women)**		-13.48	1.62	< 0.001	**-10.89**	**2.10**	**< 0.001**	-			-		
**Educational level**													
	Illiterate/primary	Ref	-	-	Ref	-	-	Ref	-	-	Ref	-	-
	Secondary/diploma	3.18	2.05	0.121	1.50	1.94	0.440	2.58	2.73	0.345	2.20	2.63	0.403
	Academic	7.74	2.56	0.003	3.84	2.48	0.121	7.82	3.92	0.046	5.59	3.80	0.142
**Marital status (unmarried)**		-4.57	2.45	0.062	-			1.39	2.93	0.633	-		
**Job status (unemployed)**		-9.25	1.74	< 0.001	-1.20	2.06	0.560	-2.14	4.88	0.660	-		
**Smoking status**													
	Non**-**smoker	Ref	-	-	Ref	-	-	Ref	-	-	Ref		
	Ex-smoker	6.62	2.79	0.018	0.43	2.65	0.870	-6.61	11.58	0.568	-		
	Smoker	7.52	2.57	0.004	-0.70	2.81	0.803	8.83	11.58	0.217	-		
**Physical activity (high)**		3.38	1.91	0.077	-			1.91	2.79	0.493	-		
**Anthropometric indices**													
	Body Mass Index, kg/m^2^	-0.80	0.17	< 0.001	**-0.51**	**0.16**	**0.002**	-0.84	0.22	< 0.001	**-0.84**	**0.22**	**< 0.001**
	Waist circumference, cm	-0.02	0.06	0.671	-			-0.06	0.10	0.576	-		
**Comorbidities (yes)**													
	Hypertension	0.21	1.78	0.905	-			-2.96	2.72	0.275	-		
	Cardiovascular disease	-4.45	1.88	0.018	**-4.03**	**1.78**	**0.024**	-5.52	2.72	0.042	-5.10	2.62	0.052
	Chronic kidney disease	-5.96	1.60	< 0.001	**-3.33**	**1.60**	**0.038**	-6.04	2.26	0.007	**-5.59**	**2.24**	**0.013**
	Dyslipidemia	-4.14	3.02	0.170	-			-4.00	4.29	0.350	-		
**Family history (yes)**													
	Diabetes	-0.43	1.93	0.822	-			1.42	2.55	0.577	-		
	Hypertension	1.49	1.98	0.452	-			2.96	2.67	0.268	-		
	Cardiovascular disease	-7.99	4.03	0.047	-5.69	3.96	0.151	-8.70	5.37	0.105	-		

**†**Crude; **††**Adjusted; **Unmarried**: Single, divorce, widow; **Unemployed**: Student, housewife, other

**Table 4:** The association between socio-behavioral factors and changes of social functioning (SF) using generalized estimating equation (GEE)

Variables		Total (*n* = 498)						Men (*n* = 236)					
		β†	SE	*P*-value	β††	SE	*P*-value	β†	SE	*P*-value	β††	SE	*P*-value
**Age (year)**		-0.08	0.09	0.360	**-**			-0.15	0.12	0.233	-		
**Sex (women)**		-5.73	1.96	0.004	-2.27	2.37	0.338	-			-		
**Educational level**													
	Illiterate/primary	Ref	-	-	Ref			Ref	-	-	Ref		
	Secondary/diploma	1.39	2.37	0.558	-			1.67	3.74	0.655	-		
	Academic	5.15	2.96	0.082	-			3.45	4.22	0.414	-		
**Marital status (unmarried)**		-1.74	2.82	0.537	-			-14.66	6.36	0.021	**-12.86**	**6.51**	**0.048**
**Job status (unemployed)**		-7.60	2.04	< 0.001	**-5.43**	**2.47**	**0.028**	-3.43	2.58	0.184	-		
**Smoking status**													
	Non**-**smoker	Ref	-	-	Ref			Ref	-	-	Ref		
	Ex-smoker	2.57	3.00	0.391	-			1.15	3.23	0.720	-		
	Smoker	-1.33	3.26	0.682	-			-3.66	3.66	0.317	-		
**Physical activity (high)**		2.84	2.29	0.216	-			5.81	3.24	0.073	-		
**Anthropometric indices**													
	Body Mass Index, kg/m^2^	-0.28	0.20	0.163	**-**			-0.15	0.33	0.633	-		
	Waist circumference, cm	0.04	0.08	0.626	-			-0.02	0.10	0.857	-		
**Comorbidities (yes)**													
	Hypertension	3.15	2.06	0.127	-			4.75	2.70	0.078	-		
	Cardiovascular disease	-4.36	2.16	0.044	-3.21	2.22	0.149	-4.08	2.99	0.172	-		
	Chronic kidney disease	-3.35	1.87	0.073	-			-2.84	2.77	0.306	-		
	Dyslipidemia	-3.57	3.51	0.309	-			-0.16	4.96	0.974	-		
**Family history (yes)**													
	Diabetes	-7.20	2.23	0.001	-4.74	2.49	0.058	-10.63	3.54	0.003	-6.81	3.99	0.087
	Hypertension	-6.41	2.30	0.005	-3.14	2.51	0.210	-12.21	3.49	< 0.001	**-8.79**	**3.81**	**0.021**
	Cardiovascular disease	-9.83	4.68	0.036	-7.68	4.88	0.116	-6.63	7.27	0.362	-		

**†**Crude; **††**Adjusted; **Unmarried**: Single, divorce, widow; **Unemployed**: Student, housewife, other

## Discussion

The study provides a comprehensive examination of the socio-behavioral determinants and their influence on the health-related quality of life of patients with T2D. The analysis of HRQoL scores, both PCS and MCS, showed a non-significant change in the total score during the 3-year longitudinal follow-up. However, the role physical subdomain of the PCS and the social functioning subdomain of the MCS showed a statistically significant change during this period.

The significant increase in the RP subdomain in diabetics may be due to the decrease in the overall prevalence of low physical activity previously reported in TLGS participants [29, 30]. The population-based cohort study by Afghan et al. that followed 3,515 participants aged 20 years and older from Phase II (2002–2005) to Phase IV (2008–2011) of the TLGS showed that the prevalence of low physical activity decreased significantly from 45.9% in Phase II to 42.6% in Phase IV [29]. This result is consistent with what we have emphasized in our work by showing the largest increase in the mean difference for the RP subdomain in total population during phases VI and VII.

In the case of the physical role, which shows a statistically significant change in women in our study, it is possible that social expectations and gender roles play a role. It is possible that the physical role of women has changed because social expectations of their tasks and activities have changed. This could be related to changes in care responsibilities, employment opportunities or other social factors.

A recent study by Kamalian et al. [31] on physical activity trends among Iranian adults aimed to fill the knowledge gap regarding recent trends in insufficient physical activity (IPA) by analyzing data from six rounds of STEPS in Iran. After adjusting for factors such as schooling, urbanization level and wealth index, the results showed a steady increase in the national prevalence of IPA over the age of 16, almost doubling from 23.1% in 2001 to 55.4% in 2016. In contrast to our results, IPA was more common in women than in men, regardless of age or province. A possible reason for these differences could be the different definition of low physical activity and the target population, which was limited to patients with diabetes in our study. Future studies are needed to investigate the exact trend of physical activity and its role in the management of diabetes in Iranian diabetic patients.

Examining the socio-behavioral determinants of RP, several factors emerged as significant contributors. In both univariable and multivariable models, sex, BMI, and the presence of CVD and CKD showed notable associations with changes in RP. Consistent with our research, prior cross-sectional studies have similarly emphasized that sex, BMI, and sufficient physical activity are significant factors associated with PCS [32–34]. A study from Stanford also showed that in diabetics, the presence of obesity significantly impairs HRQoL [35]. Our study reinforces the evidence, as these factors maintained their statistical significance as correlates throughout the 3-year follow-up period.

The significant decrease in the SF subdomain of MCS in diabetics could be attributed to the increased burden of mental disorders in Iranian adults [36]. This could be due to the potential impact of mental disorders on social functioning [37]. A 2017 study aimed to provide a comprehensive overview of the adaptive social functioning of individuals with depressive and anxiety disorders, even after achieving remission. The results showed a worrying escalation trend in social dysfunction across all patient groups, with individuals with comorbid anxiety and depressive disorders showing the most severe impairment, followed by individuals with depressive and anxiety disorders. Affective indicators showed the largest effect sizes, illustrating their pronounced impact. Remarkably, the impairments in social functioning also persisted in the remitted patients [37]. These results emphasize the importance of managing patients’ mental health in order to achieve a better quality of life. A recent systematic review delves into the association between psychological constructs of well-being and medical outcomes in patients with diabetes [38]. The analysis included well-being interventions such as positive psychology, mindfulness-based and resilience-based interventions. Most studies highlighted positive outcomes of interventions, which included improved glycemic control and effective blood pressure management [38].

On the other hand, the decline in the social functioning subdomain of the MCS in men could be influenced by different societal expectations and coping mechanisms. Societal norms and expectations regarding sex roles and behavior may influence how men and women with diabetes perceive and participate in social interactions. In addition, men and women may use different coping strategies to deal with the challenges of living with diabetes. These strategies could affect their social functioning and interactions in different ways.

The socio-behavioral determinants of social functioning (SF) showed nuanced associations. Unemployment was found to be a significant factor affecting SF changes in all patients, highlighting the influence of job status on social aspects of HRQoL [39]. In men, marital status and family history of diabetes and hypertension played a central role in shaping the SF. Marital status can have a significant impact on men’s social functioning due to the traditional societal expectations and roles associated with marriage. In addition, the results of Manjunath at al.‘s study of patients with T2D in India showed that men and those who were currently married had statistically significantly better quality of life [40]. For men, marriage can bring changes in terms of social responsibilities, support systems and social interactions. In addition, societal norms and expectations regarding the role of husbands and fathers within the family structure can influence how marital status affects men’s social functioning.

Regarding family history of diabetes and hypertension, these factors may have sex-specific effects on social functioning due to potential differences in health-related behaviors and coping mechanisms between men and women. The findings underscore the need to consider not only individual health behaviors but also the broader social and family context when developing interventions to improve social functioning in T2D patients.

### Implications

The findings emphasize the importance of tailoring interventions to the individual needs of patients with T2D, taking into account sex-specific patterns in HRQoL changes. Integrating educational initiatives that focus specifically on BMI management and prevention of comorbidities such as CVD and CKD could have a positive impact on role physical outcomes. In addition, addressing social determinants, such as unemployment and marital status is critical to improving social functioning and contributes to a more holistic approach to patient-centered care.

### Limitations and future directions

Even though the study provides valuable insights, certain limitations must be acknowledged. The assessment period of three years may not capture all dynamic changes in socio-behavioral determinants. The study could benefit from a specific diabetes-specific QoL questionnaire [41]. Arditi et al. [42] investigated the health status and quality of life of diabetes patients. In the study, univariate and multivariate regression analyses were performed on health status (PCS and MCS) and diabetes-specific QoL (ADDQoL score). The specific areas of life with the lowest quality of life scores included freedom to eat, sex life, and freedom to drink. Notably, older age was independently associated with better mental health and QoL, while lower income correlated with poorer physical and mental health, and QoL. Longer duration of diabetes (over 10 years), insulin treatment, and complications were associated with lower QoL and physical health. Future research could benefit from a longer follow-up period and investigate additional factors, such as cultural influences, that may contribute to variations in HRQoL outcomes among T2D patients.

## Conclusion

In conclusion, the study reveals the intricate web of socio-behavioral determinants that influence the HRQoL of patients with T2D. By recognizing the sex-specific patterns in HRQoL changes and understanding the multifaceted nature of factors such as BMI, CVD and CKD, healthcare professionals can develop targeted interventions that go beyond traditional diabetes management. The findings underscore the importance of patient-centered care, acknowledging the unique needs of patients and tailoring interventions to foster holistic well-being in the journey of living with type 2 diabetes.

## Data Availability

The datasets used and/or analysed during the current study are available from the corresponding author on reasonable request.
